# Duration of Absence from Work Is Related to Psychopathology, Personality, and Sociodemographic Variables in a Longitudinal Cohort

**DOI:** 10.3389/fpsyt.2017.00252

**Published:** 2017-11-29

**Authors:** Alex Gamma, Roman Schleifer, Ingeborg Warnke, Vladeta Ajdacic-Gross, Wulf Rössler, Jules Angst, Michael Liebrenz

**Affiliations:** ^1^Department of Forensic Psychiatry, Institute of Forensic Medicine, University of Bern, Bern, Switzerland; ^2^Department of Psychiatry, Psychotherapy and Psychosomatics, Psychiatric Hospital, University of Zurich, Zurich, Switzerland; ^3^Laboratory of Neuroscience (LIM 27), Department and Institute of Psychiatry, Faculty of Medicine, University of Sao Paulo, Sao Paulo, Brazil

**Keywords:** work participation, psychopathology, longitudinal, Switzerland, epidemiology

## Abstract

**Objective:**

To examine, in a non-clinical sample, the association of psychopathology, personality, sociodemographic information, and psychosocial indicators of non-occupational functioning with the duration of absence from work in the past 12 months.

**Method:**

A longitudinal community cohort of 591 adults from Switzerland was analyzed using multilevel ordered logistic regression, with several alternative models as robustness checks. Psychopathology was assessed using the total score (Global Severity Index) of the Symptom Check List-90 Revised.

**Results:**

The highest psychopathology levels were associated with absences of 3 or more week duration, largely independently of age. Extraversion and being divorced, widowed or separated also corresponded with longer absences from work in some analyses. No effect of sex was found. Most effects tested were not statistically significant and estimates showed large uncertainty.

**Conclusion:**

Although tentative, our results suggest a possible influence of psychopathology on work participation. It may thus be desirable in insurance-medical appraisals of work ability to include instruments for measuring psychopathology.

## Introduction

Psychiatric disorders are associated with a higher risk of sick leave from work (1, 2), but other risk factors such as social functioning, personality, and environment are also increasingly being studied. The World Health Organization has proposed the International Classification of Functioning, Disability, and Health (ICF) (3) as an integrated framework to conceptually organize this research. In explaining disorders of work participation, it distinguishes between symptoms of illness, e.g., psychopathology, and disorders of capacity, e.g., limitations in executing activities (4). The ICF has gained importance in the contexts of insurance medical examination of work ability and of rehabilitation (5).

With regard to illness symptoms, Baron and Linden (4) have failed to find a relationship between general psychopathology and duration of sick leave in a German sample of inpatients with chronic neurotic and affective disorders. They did, however, find a link between a broad range of impairments of capacity/activity and sick leave. Based on these results, it has been proposed to give precedence to disorders of capacity when assessing work ability in an insurance medical context (6).

However, these connections between psychopathology and work participation may be different in the general population, where symptom load and levels of chronification are lower. Also, Baron and Linden have used absolute durations of sick leave with no relativization to subjects’ age or to the amount of time during which they were part of the labor force. Some form of standardization is desirable because a sick leave of 2 years in a 25-year-old patient may be something very different than the same duration of sick leave in a 55-year-old patient.

With regard to disorders of capacity, Muschalla et al. (7) found higher levels of health-related work problems to be accompanied by higher impairment in non-occupational areas in subjects recruited from waiting rooms of primary care physicians. Non-occupational functioning included daily duties, social activities, close personal relationships and sexual activities. However, the possible role of psychopathology was not examined.

This study therefore primarily aimed at examining, in a non-clinical, longitudinal community sample, the relationship between work participation and (i) general psychopathology [using the same rating instrument as (4)] and (ii) non-occupational functioning, including social activities and relationship quality. In addition, possible contributions of age, sex, and personality were investigated. The analysis focused on the effects of psychopathology on duration of absence from work, allowing for an interaction with age and thus the possibility that psychopathology could differentially affect work participation at different ages.

## Materials and Methods

### Subjects

Subjects were participants in the Swiss “Zurich study,” a longitudinal study of somatic and psychiatric complaints in adults aged 20/21 to 49/50 years. Data were collected using a comprehensive structured interview (“SPIKE”) applied by trained interviewers in participants’ homes. Seven interview waves have been conducted so far, in the years 1979, 1981, 1986, 1988, 1993, 1999, and 2008. For the first interview wave in 1979, 591 participants were selected from a larger representative screening population consisting of all male conscripts to the army (age 19, *N* = 2,201) and all women enrolled in the electoral register (age 20, *N* = 2,346) in the canton of Zurich, Switzerland.

Sample selection followed a stratified sampling procedure, whereby the sample was enriched with cases at risk for the development of psychiatric and/or somatic syndromes. High-risk subjects were defined by initial Symptom Check List-90 Revised (SCL-90R) total scores [Global Severity Index (GSI) scores] above the 85th percentile and make up one-third of the sample, while the low-risk group was randomly selected from subjects scoring below the 85th percentile in the SCL-90R. The resulting sample consisted of 591 subjects (292 men, 299 women). All participants provided written informed consent. The study was approved by the institutional review board of the University of Zurich.

### Assessments

#### Outcome

The outcome variable was the duration of absence from work during the past 12 months. Importantly, this question was not asked in the same way across all interviews. The first interview in 1979 only asked about absence from “work” (presumably paid work, except holidays) and did not explicitly consider absence from housework or school. The interviews in 1981 and 1986 asked about absence from paid work or from non-paid daily housework (for housewives) or from school/lectures (for students). Subjects were explicitly instructed to ignore planned absences such as holidays, planned breaks from housework, or school holidays/semester breaks. In the remaining interview years (1988–1999), the question referred to the number of days of sick leave, and again did not explicitly consider housework or school. The response format up to and including 1986 was a Likert scale from 1 to 6 (1 = no absence, 2 = 1–2 days, 3 = 3–6 days, 4 = 1–2 weeks, 5 = 3–4 weeks, 6 = more than 4 weeks). After that, the number of days was recorded as a continuous variable. The variable was not available for 2008, which is therefore excluded from all analyses. The outcome was reduced to three response categories (no absence, 1–14 days, ≥3 weeks) for statistical analysis (see Statistics). One thing to note about the Likert scale used until 1986 is that durations of 2–3 weeks are not accounted for, as the scale skips from “1–2” to “3–4 weeks.” It is not clear how respondents handled that gap, but it seems most likely that they would choose one of the adjacent response categories.

#### General Psychopathology (SCL-90R)

As a measure of general psychopathology, we used the SCL-90R questionnaire (8). The SCL-90-R is a self-report inventory of 90 symptoms that characterize various psychiatric and psychosomatic conditions. The degree to which each symptom has been present in the past month was rated on a scale from 0 to 4 (0 = not at all, 1 = a little bit, 2 = moderately, 3 = quite a bit, 4 = extremely). There are nine subscales (which we did not use in this study) as well as a total score called GSI. The GSI is the mean of all item scores.

#### Personality (FPI)

We assessed personality traits using the “Freiburg Personality Inventory” (FPI). The FPI comprises 212 self-rated yes/no items grouped into nine primary factors (nervousness, spontaneous aggression, depressiveness, irritability, sociability, resilience, striving for dominance, inhibition, and openness) (9). For analysis, we used three secondary factors (aggressiveness/irritability, extraversion, and neuroticism/vegetative lability) derived from a factor analysis of large samples, as described elsewhere (10). Data were collected in two out of the seven interviews only: at ages 29/30 (in 1988) and 34/35 (in 1993).

#### Occupational and Social Functioning

The SPIKE contains several questions indicating an individual’s functioning in several life domains during the past 12 months. We used the following items: jobless (yes/no), conflicts at work (yes/no), conflict with friends (none/has happened/occasional/frequent/termination of relationship), marital status (single/married/divorced, separated, and widowed), conflict with partner (none/has happened/occasional/frequent/termination of relationship), increasing difficulties with partner (yes/no), improvement of relationship with partner (yes/no), number of meetings with members of the opposite sex (none/1 meeting per month/1 meeting every 2 weeks/1–2 meetings per week/>2 meetings per week), number of close friends (0/1/2–4/5–8/9–16/16+), lives alone (yes/no).

### Statistics

To address the main question of the relationship between duration of absence from work and psychopathology, personality, age, and occupational and social functioning, we used multilevel multinomial logistic regression with shared random intercepts. The categories of the outcome were reduced from six to three levels of duration: no absence/1–14 days/>3 weeks. Although such reduction is generally advised against to avoid loss of information, we deemed it necessary to keep the model estimable, since the highest three levels had small cell sizes. Retaining three levels still allowed to detect a possible “dose–response” effect.

For analysis of the FPI, we took the mean of the two measurements from 1988 and 1993 and imputed them for all the missing interview years, assuming that personality is a relatively stable aspect of an individual. Indeed, scores of these two measurements showed reasonably good agreement, with correlations ranging from rho = 0.6 to 0.8. Nevertheless, applying these means to a study period spanning 30 years remains a risky operation that will introduce substantial uncertainty in the relevant estimates. Therefore, we included among the robustness checks an analysis without any FPI predictors.

FPI and GSI were normalized to make their effects comparable and more easy to interpret. The GSI was centered and standardized at the mean and SD of a low-risk subject in the first interview. FPI scores were centered and standardized at the global mean and SD (data were available only in 1988 and 1993).

No model reduction was performed as predictive optimization was not the aim and because such procedures tend to inflate false-positive rates beyond nominal levels.

We ran five models in total (Table 1). The main model included the three levels of absence from work as outcome and the full set of predictors as described earlier, but excluded the year 1988, for which many of the predictors had been omitted in an attempt to keep the interview at a length still manageable for participants. As robustness checks, we computed four additional models whose results are presented in the Supplementary Material. These are also summarized in Table 1. Two of the alternative models included the data from 1988.

**Table 1:** Overview of models.

	Model 1 (main)	Model 2 (incl. 1988 data)	Model 3 (only GSI and year)	Model 4 (no FPI)	Model 5 (all outcome categories)
Type	Multilevel multinomial logistic	Multilevel multinomial logistic	Multilevel multinomial logistic	Multilevel multinomial logistic	Multilevel multinomial logistic

Sample	Excl. 1988	Incl. 1988	Incl. 1988	Excl. 1988	Excl. 1988

Dependent variables	3 levels	3 levels	3 levels	3 levels	6 levels

Predictors	Sex, GSI, year, GSI × year, FPI (3 variables), conflicts at work, marital status, conflicts with friends, conflicts with partner, increasing difficulties with partner, improvements with partner, *N* meetings with opposite sex, living alone, *N* close friends, and jobless	Without: *N* close friends, conflicts with friends, conflicts with partner, number of meetings with opposite sex (all missing in 1988)	Only: GSI, year and GSI × year	Without: FPI (personality) variables	Like model 1

*FPI, Freiburg Personality Inventory; GSI, Global Severity Index*.

Technically, all models were multilevel multinomial logistic regressions. They were run in a structural equation framework via Stata’s *gsem* command, since no dedicated command for multilevel multinomial logistic models is currently available in Stata. Measures of explained variance (*R*^2^ or Pseudo-*R*^2^) were not available with this command. To nevertheless get a sense of the variance explained, we ran the main model as a conventional ordered logistic regression (Stata’s *ologit* command) model with robust standard errors corrected for the clustering of observations within subjects.

Analyses were performed in Stata 14.2 for Mac (11).

## Results

### Descriptive Statistics

Table 2 shows predictor frequencies and means, broken down by outcome category (duration of absence from work). Level of schooling, which was not included in models, is also provided. For this data summary, variables were collapsed across interview years: the highest category was chosen for the outcome, the median or mean for the predictors.

**Table 2:** Sociodemographic variables and predictors by level of absence from work in the previous 12 months.

		No absence^a^		1–2 days^a^		3–6 days^a^		1–2 weeks^a^		3–4 weeks^a^		>4 weeks^a^		Total	
	Median^b^	*N*	%	*N*	%	*N*	%	*N*	%	*N*	%	*N*	%	*N*	%
*N*	–	125	21.2	91	15.4	137	23.2	120	20.3	55	9.3	63	10.7	951	100
Male	1	63	50.4	47	51.6	76	55.5	49	40.8	28	50.9	29	46	292	49.4
**Level of schooling**															
Middle school (low)	1	11	8.8	3	3.3	7	5.11	5	4.17	3	5.45	5	7.94	34	5.75
Middle school (medium)	3	40	32	23	25.27	32	23.36	32	26.67	16	29.09	24	38.1	167	28.26
Middle school (high)	4	37	29.6	39	42.86	54	39.42	39	32.5	21	38.18	15	23.81	205	34.69
Middle school (other)	7	18	14.4	7	7.69	18	13.14	9	7.5	2	3.64	8	12.7	62	10.49
High school	9	14	11.2	16	17.58	23	16.79	28	23.33	11	20	7	11.11	99	16.75
Missing		5	4	3	3.3	3	2.19	7	5.83	2	3.64	4	6.35	24	4.06
**Conflicts at work**															
No	0	101	80.8	79	86.8	119	86.9	101	84.2	46	83.6	46	73	492	83.2
	0.5	12	9.6	4	4.4	9	6.6	6	5	2	3.6	7	11.1	40	6.8
Yes	1	12	9.6	8	8.8	9	6.6	13	10.8	7	12.7	10	15.9	59	10
**Marital status**															
Single	1	89	71.2	56	61.5	75	54.7	68	56.7	28	50.9	29	46	345	58.4
	1.5	9	7.2	3	3.3	7	5.1	6	5	5	9.1	2	3.2	32	5.4
Married	2	26	20.8	31	34.1	52	38	39	32.5	21	38.2	28	44.4	197	33.3
	2.5	1	0.8	1	1.1	1	0.7	3	2.5	0	0	0	0	6	1
Divorced/widowed/separated	3	0	0	0	0	2	1.5	4	3.3	1	1.8	4	6.3	11	1.9
**Friendship conflicts**															
None	1	76	60.8	63	69.2	95	69.3	89	74.2	41	74.5	38	60.3	402	68
	1.5	22	17.6	12	13.2	24	17.5	15	12.5	5	9.1	11	17.5	89	15.1
Have occurred	2	22	17.6	12	13.2	13	9.5	16	13.3	8	14.5	13	20.6	84	14.2
	2.5	1	0.8	0	0	2	1.5	0	0	0	0	1	1.6	4	0.7
Occasionally	3	3	2.4	4	4.4	3	2.2	0	0	1	1.8	0	0	11	1.9
Termination of relationship	5	1	0.8	0	0	0	0	0	0	0	0	0	0	1	0.2
**Relationship conflicts**															
None	1	84	67.2	56	61.5	68	49.6	61	50.8	32	58.2	29	46	330	55.8
	1.5	11	8.8	10	11	23	16.8	14	11.7	3	5.5	16	25.4	77	13
Have occurred	2	24	19.2	20	22	39	28.5	37	30.8	15	27.3	10	15.9	145	24.5
	2.5	2	1.6	2	2.2	5	3.6	4	3.3	0	0	5	7.9	18	3
Occasionally	3	2	1.6	1	1.1	2	1.5	4	3.3	3	5.5	2	3.2	14	2.4
	3.5	0	0	1	1.1	0	0	0	0	1	1.8	0	0	2	0.3
Frequently	4	1	0.8	0	0	0	0	0	0	0	0	1	1.6	2	0.3
Termination of relationship	5	1	0.8	1	1.1	0	0	0	0	1	1.8	0	0	3	0.5
**Increasing difficulties with partner**															
No	0	104	83.2	84	92.3	126	92	109	90.8	47	85.5	55	87.3	525	88.8
	0.5	8	6.4	2	2.2	5	3.6	6	5	4	7.3	4	6.3	29	4.9
Yes	1	13	10.4	5	5.5	6	4.4	5	4.2	4	7.3	4	6.3	37	6.3
**Improved relationship with partner**															
No	0	100	80	81	89	123	89.8	110	91.7	51	92.7	56	88.9	521	88.2
	0.5	10	8	3	3.3	4	2.9	6	5	1	1.8	4	6.3	28	4.7
Yes	1	15	12	7	7.7	10	7.3	4	3.3	3	5.5	3	4.8	42	7.1
***N* meetings with persons of opposite sex**															
≥2 per week	1	61	49.2	40	44	68	50	52	43.7	24	44.4	27	42.9	272	46.3
	1.5	6	4.8	2	2.2	6	4.4	8	6.7	4	7.4	7	11.1	33	5.6
1–2 per week	2	17	13.7	16	17.6	30	22.1	27	22.7	16	29.6	14	22.2	120	20.4
	2.5	2	1.6	6	6.6	4	2.9	3	2.5	1	1.9	2	3.2	18	3.1
1 every 2 weeks	3	14	11.3	10	11	14	10.3	9	7.6	3	5.6	6	9.5	56	9.5
	3.5	1	0.8	2	2.2	2	1.5	5	4.2	3	5.6	0	0	13	2.2
1 per month	4	10	8.1	9	9.9	6	4.4	10	8.4	2	3.7	6	9.5	43	7.3
	4.5	1	0.8	0	0	1	0.7	1	0.8	0	0	0	0	3	0.5
None	5	12	9.7	6	6.6	5	3.7	4	3.4	1	1.9	1	1.6	29	4.9
**Lives alone**															
No	0	104	83.2	75	82.4	122	89.1	102	85	50	90.9	54	85.7	507	85.8
	0.5	11	8.8	6	6.6	4	2.9	6	5	1	1.8	2	3.2	30	5.1
Yes	1	10	8	10	11	11	8	12	10	4	7.3	7	11.1	54	9.1
**Number of close friends**															
>16	1	3	2.4	0	0	0	0	0	0	2	3.6	1	1.6	6	1
	1.5	1	0.8	0	0	0	0	0	0	0	0	1	1.6	2	0.3
9–16	2	7	5.6	4	4.4	9	6.6	4	3.3	3	5.5	1	1.6	28	4.7
	2.5	2	1.6	3	3.3	7	5.1	5	4.2	2	3.6	5	7.9	24	4.1
5–8	3	46	36.8	30	33	53	38.7	39	32.5	20	36.4	21	33.3	209	35.4
	3.5	12	9.6	13	14.3	15	10.9	19	15.8	4	7.3	10	15.9	73	12.4
2–4	4	46	36.8	39	42.9	52	38	51	42.5	19	34.5	20	31.7	227	38.4
	4.5	2	1.6	0	0	0	0	2	1.7	1	1.8	0	0	5	0.8
1	5	6	4.8	2	2.2	0	0	0	0	3	5.5	3	4.8	14	2.4
0	6	0	0	0	0	1	0.7	0	0	1	1.8	1	1.6	3	0.5
**Jobless**															
No	0	122	97.6	89	97.8	137	100	118	98.3	55	100	61	96.8	582	98.5
	0.5	2	1.6	2	2.2	0	0	2	1.7	0	0	2	3.2	8	1.4
Yes	1	1	0.8	0	0	0	0	0	0	0	0	0	0	1	0.2

		**Mean**	**SD**	**Mean**	**SD**	**Mean**	**SD**	**Mean**	**SD**	**Mean**	**SD**	**Mean**	**SD**	**Mean**	**SD**

Psychopathology score (GSI)^c^	–	0.29	0.75	0.44	0.74	0.39	0.68	0.57	0.76	0.7	0.95	0.54	0.78	0.46	0.77
Aggressiveness/irritability (FPI)^d^	–	2.17	0.77	2.57	1.02	2.52	0.92	2.84	1.05	2.76	1.01	2.69	1.1	2.61	1
Extraversion (FPI)^d^	–	2.32	0.98	2.49	1.02	2.54	1.03	2.43	0.96	2.53	1.04	2.75	0.98	2.51	1
Neuroticism/vegetative lability (FPI)^d^	–	1.91	0.78	2.28	1.04	2.28	0.91	2.69	1.02	2.67	1.13	2.52	0.93	2.41	1

*^a^This being a longitudinal study, outcomes and predictors had to be collapsed across interview years. For the outcome variable, the highest category across interviews was chosen. For the predictors, it was the median (for categorical variables) or the mean (for continuous variables)*.

*^b^The median of categorical predictors across interviews can have values in-between the original categories. These are not labeled*.

*^c^The GSI was centered and standardized at the mean and SD of a low-risk subject in the first interview*.

*^d^FPI scores were centered and standardized at the global mean and SD (data available only for the fourth and fifth interview)*.

*FPI, Freiburg Personality Inventory; GSI, Global Severity Index (total score of the Symptom Check List-90 Revised questionnaire)*.

**Figure 1:**
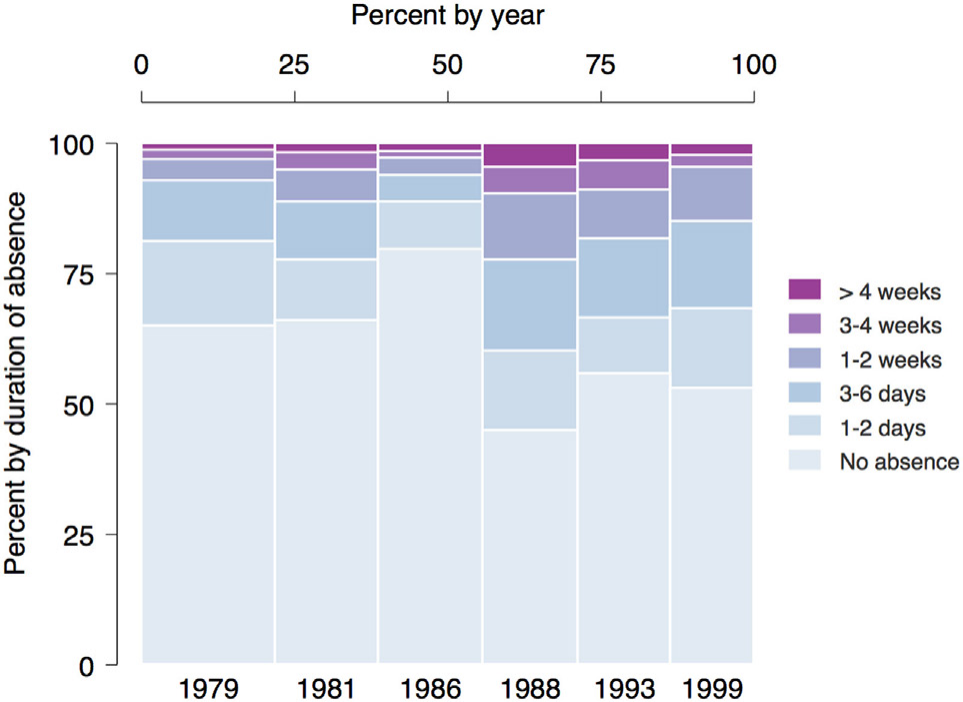
Spineplot showing duration of absence from work by interview year. It can be read like a stacked bar graph with the added benefit that the width of the bars for each year is proportional to the number of subjects in that year. Thus, the size of each cell faithfully represents the overall proportion of subjects in that cell. It can be seen that the proportion of subjects without absence from work declines after 1986 (ages 27/28), while the proportion of those with absences of 3 or more weeks simultaneously increases.

Figure 1 additionally shows a breakdown of outcome categories by interview year. The percentage of subjects reporting no absence from work in the previous 12 months was around 65% in 1979 and 1981 (ages 19–22), jumped to over 75% in 1986 (ages 27/28) and then dropped down to around 50% for the remaining years. Long absences of 3 or more weeks were particularly frequent in 1988 and 1993 (ages 29–35).

**Figure 2:**
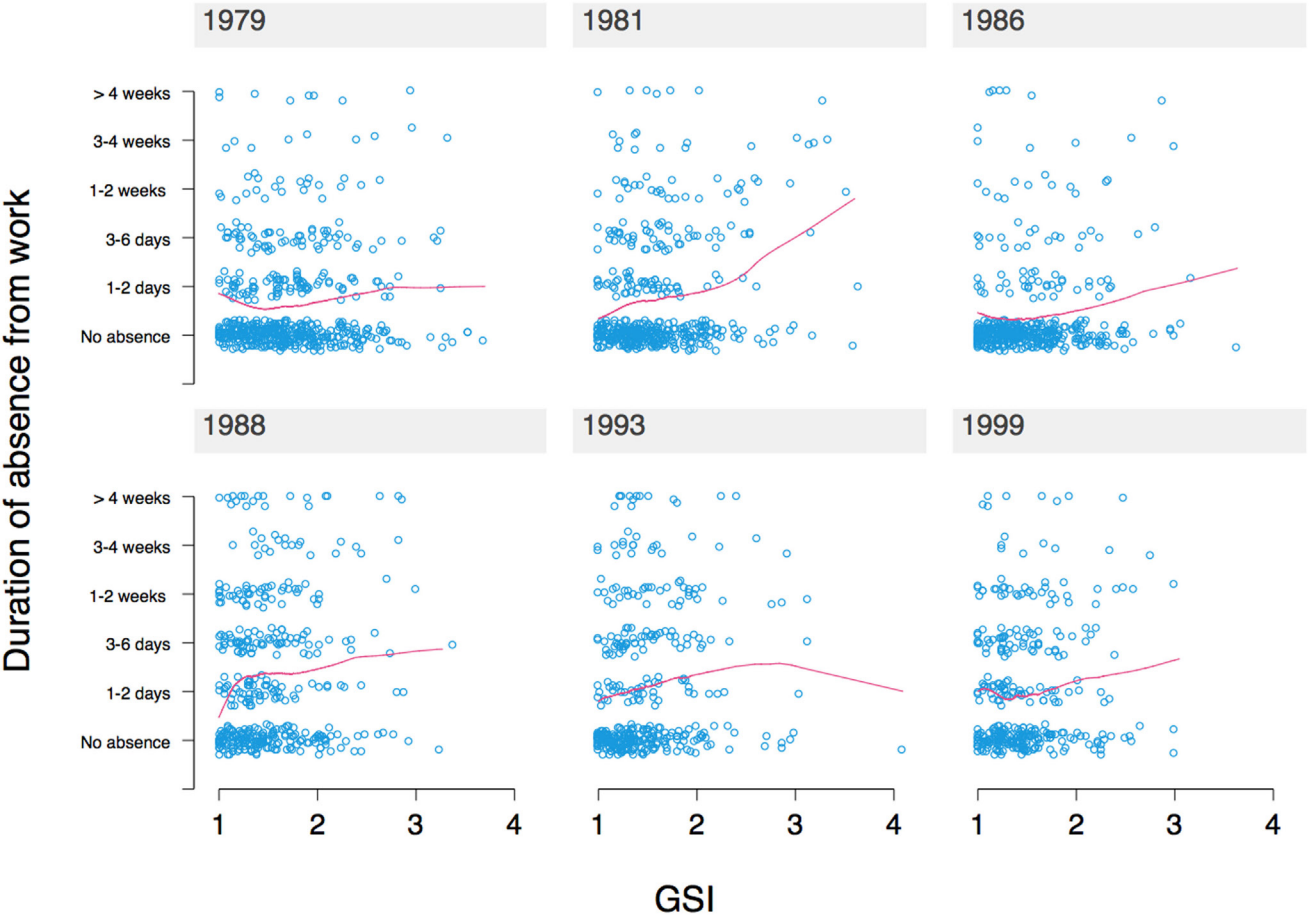
Dependency of the duration of absence from work in the previous 12 months on psychopathology score [Global Severity Index (GSI)], broken down by interview year. Subjects are ages 20/21 in 1979. The red lines are fitted curves estimated using locally weighted scatterplot smoothing (LOESS).

Figure 2 shows the dependency of duration of absence on level of psychopathology by interview year. There is a positive association for all years, which is most prominent for 1981, when subjects were aged 22/23 years.

### Regression Effects on Absence from Work

**Figure 3:**
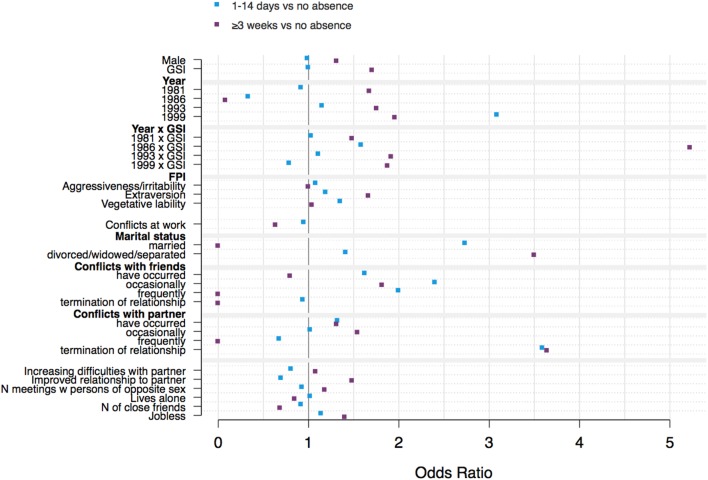
Statistical effects (ORs) of model predictors on duration of absence from work on the odds ratio scale. Confidence intervals are too large to be shown, indicating large uncertainty in estimates. (Figure S1 in Supplementary Material shows the same results plotted on the log-odds scale and including confidence intervals.) Shown in blue are predictor effects on the probability of switching from no absence to up to 2 weeks of absence. Shown in purple are effects on the probability of switching from no to 3 and more weeks of absence.

**Figure 4:**
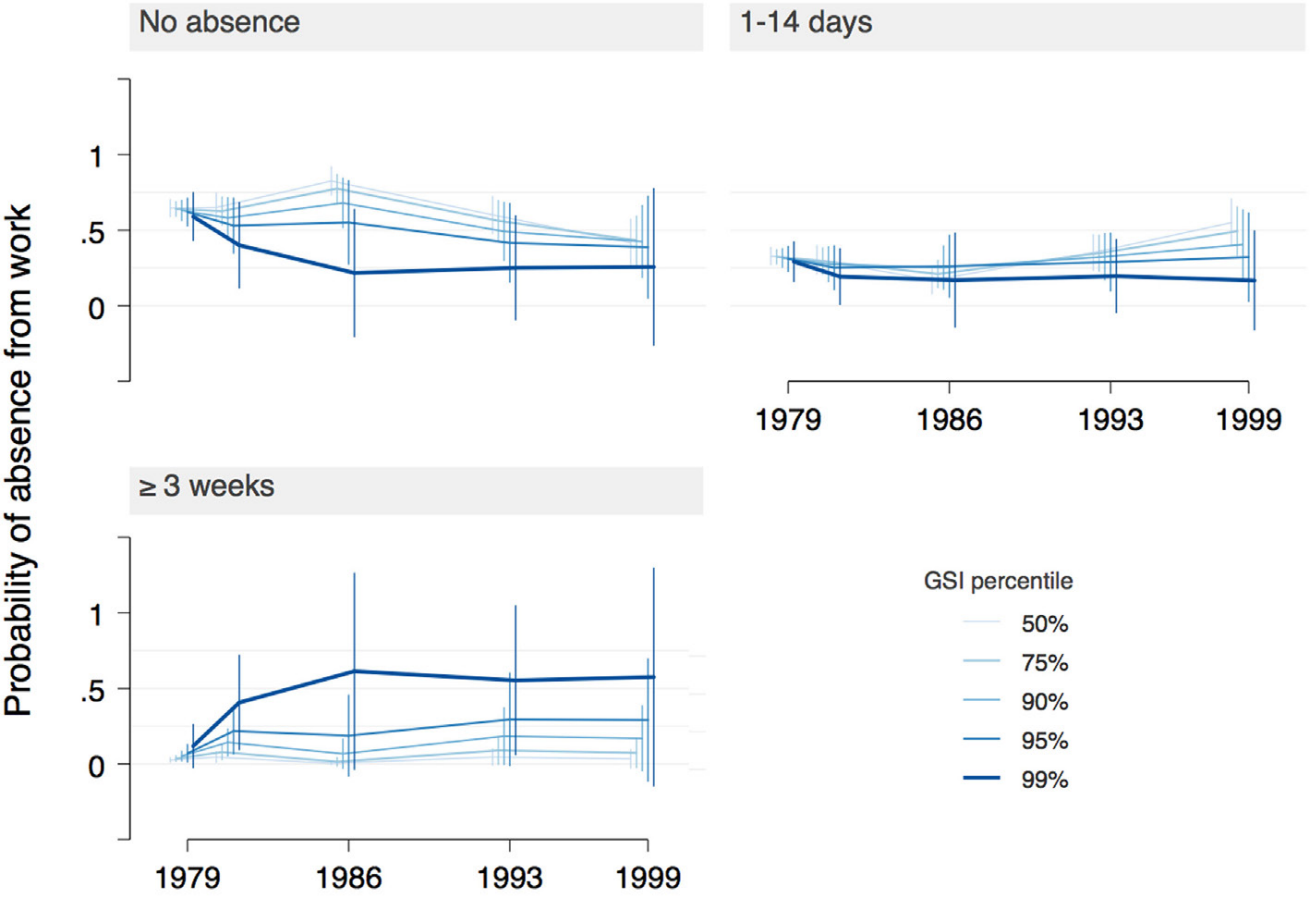
Main model: probability of three levels of absence from work (none, 1–14 days, 3 or more weeks) across interview years, as a function of increasing psychopathology levels [Global Severity Index (GSI)]. The highest percentile of GSI was associated with fewer years with no absence and more years with 3 or more weeks of absence, particularly at and after ages 27/28. However, confidence intervals are large, indicating high uncertainty of estimates.

Models in general showed mostly small effects and a few large effects with high uncertainty. Few effects were statistically significant. The following results describe the main model (Figures 3 and 4; Tables S1 and S2 in Supplementary Material). Where other models deviate in a way that might lead to different conclusions, it will be noted.

General psychopathology score (GSI) was associated with long absences from work. A 1 SD increase in GSI score relative to the mean for a low-risk subject was related to 30% higher odds (~5% increase in probability) of being absent from work for 3 or more weeks.

A more nuanced picture emerges from the margins plot in Figure 4. For most participants (GSI below 90th percentile) the probability of no absences peaked at ages 27/28 (probability = 75%; ORs < 0.5 compared with age 19) and was lowest at ages 49/50 (~45%, ORs > 1.5 compared with age 19). Absences of 1–14 days duration became substantially more likely at later ages (probability ~50%), while absences of 3 weeks or more became slightly more likely with age. For the 1% with highest psychopathology scores, years without absences became less likely up to ages 27/28 (probability ~25%), while during the same interval the frequency of absences of 3 or more weeks increased steeply and remained high (probability ~60%). The effects for GSI scores in-between the median and the highest percentile line up in a “dose–response” relationship. Estimates for the top 1% of GSI have very high uncertainty, however.

**Figure 5:**
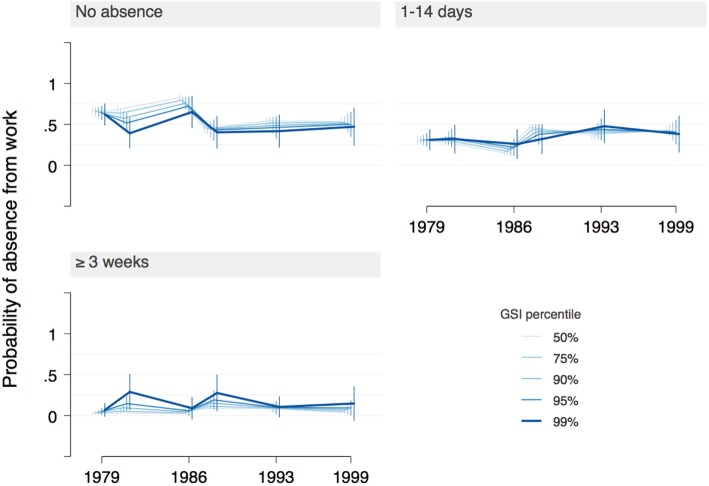
Model 2: probability of three levels of absence from work (none, 1–14 days, 3 or more weeks) across interview years, as a function of increasing psychopathology levels [Global Severity Index (GSI)]. The statistical effect of the highest GSI percentile on long absences from work is less pronounced than in the main model (Figure 4). Confidence intervals are smaller than in the main model, indicating less uncertainty of estimates.

With regard to the highest percentile of GSI, the other models differed quite markedly from the main model. Models 2–4 (Figures S4, S6, and S8 in Supplementary Material) do not show the steep increase in absences of 3 or more weeks in 1986, but rather a small decrease compared with 1981. They do, however, show such an increase for 1988 (models 2–3) or later (model 4), which, nevertheless, does not reach the high values of the main model. Furthermore, the main model’s high-GSI-related decrease of years without absences both over time as well as compared with the lower GSI percentiles was less pronounced in the alternative models. Model 2, which includes the data from 1988, is shown as an example in Figure 5 (reproduced as Figure S4 in Supplementary Material).

Of the three personality subscales—aggressiveness/irritability, extraversion, neuroticism/vegetative lability—only extraversion was associated with longer absences from work (ORs = 1.7). However, model 2 that included data from 1988 showed positive associations with all subscales in the range of OR 1.1–1.7.

Conflicts at work were negatively (but not statistically significantly) related to longer durations of absence from work, which did not seem particularly plausible. Indeed, the model including 1988 data diverged and showed positive effects in the range of OR = 1.2–1.5 (for short, 1- to 14-day absences and longer, 3 or more week absences, respectively).

Compared with being single, being divorced, widowed or separated was positively associated with duration of absence from work. The latter effect was particularly pronounced for absences of 3 or more weeks (OR = 3.5). However, both confidence intervals included 0. The effect of being married on longer absences could not be properly estimated (huge confidence intervals). However, the model including 1988 data showed negative associations with duration of absence with ORs of around 0.75.

The effects of conflict with friends or partners were inconsistent, and some could not be estimated (huge confidence intervals). Other predictors, in particular sex, showed no clear associations with work participation.

The Pseudo-*R*^2^ for the ordered logistic regression with the predictors from the main model was 0.07.

## Discussion

In a community sample of 591 adults, we found some evidence that long durations of absence from work are related to higher psychopathology, higher extraversion, older age, and being divorced, widowed or separated. Most results, however, were not statistically significant, and dose–response relationships (larger effects on a given outcome level by higher predictor levels, or larger effects on higher outcome levels by a given predictor level) were not always consistently observed. The models also explained no more than 10% of the variance in the data.

We found some support for the idea that psychopathology interferes with work participation in a non-clinical sample, and it was particularly the highest levels that produced noticeable effects. The contrast to Baron and Linden’s (4) null result (no association of psychopathology and sick leave found) may be due to the smaller proportion of chronified disorders present in our sample or it may simply reflect a lack of statistical power in their study. It should also be noted that an examination of dropouts in the Zurich study at age 40/41 showed that they preferentially occurred at the extremes of the GSI distribution (12), so that the true effect of psychopathology on work participation at higher ages might be larger than what is reported here.

There was little support for the effect of psychopathology being different at different ages. However, being statistical null results, this should be interpreted as a failure to find an effect, not as a confirmation of the absence of an effect.

FPI extraversion was consistently associated with longer absences. This is partly in line with Furnham and Miller’s (13) report of a higher number of periods of sick leave in extraverted subjects, particularly younger ones. However, the actual number of days of sick leave per annum was not found to be statistically significant, which is potentially at odds with our findings. Also, a large study from the Netherlands found results opposite to ours, i.e., a negative association of extraversion with duration of absence from work (14). Among the possible reasons for this difference are: differences in study samples (for example, the mean age in the Dutch study was around 40 years, which is the maximum age in our study), differences in statistical analysis (cross-sectional vs. longitudinal), and different constructs of extraversion applied (NEO-FFI in the Dutch vs. FPI in our study). Finally, results from any of the studies could be spurious.

A link between conflicts at work and sick leave would have been plausible, but could not be confirmed in this study, although an analysis in a different sample from the same catchment area did report such a link (15). Our study may have lacked the statistical power to detect the effect, or it might be truly absent if, for example, employees consciously try to avoid absences for fear of losing their job, even in case of interpersonal conflicts. A further possibility is that there is some interaction or collinearity with another predictor variable.

Finding longer absences in divorced, separated and widowed individuals was again plausible, as divorce, separation and death of a partner can cause considerable emotional stress and disrupt the ability to function in several domains of life.

We were unable to find effects of sex on work participation. The literature on this subject contains both positive and negative results. Hensing et al. (2) reported more spells of absence in women, which, however, were shorter than those in men. Laitinen-Krispijn and Bijl (1) reported more frequent absences in men. Muschalla et al. (7) did not find sex differences with regard to health-related work participation restrictions. According to official Swiss government statistics for the years 2010–2015, women were somewhat more frequently absent from work than men, while in the two decades before that, men had longer absences (16). Thus, overall there does not seem to be a gender effect of a size large enough to manifest in most contexts.

This study has several limitations. Apart from the relatively large uncertainty in effect estimates, the biggest single limitation of this study is probably the inconsistency in the definition of the outcome variable across time, which renders predictor effects less comparable between interview years. In 1979, work participation only referred to paid work, not to school or housework. In the subsequent two interviews (1981, 1986), school/university and housework were explicitly included. This difference may partly explain why there was no GSI effect on work participation in 1979 compared with later interviews. On the other hand, there was no obvious difference in outcome proportions in 1979 compared with other years.

Another change in the outcome variable occurred after 1986, when the response format switched from a Likert to a continuous scale recording the number of days of sick leave, and again did not explicitly consider housework or school. These changes in meaning of the outcome variable confound the interpretation of predictor effects to an unknown extent.

There is one peculiar error in the Likert scale used from 1979 to 1986: it ignores durations of absence from work of 2–3 weeks (the scale skips from 1–2 to 3–4 weeks). The consequences of this are not entirely clear, but since the category of 3 or more weeks absence has the smallest sample size, they depend most on how many respondents with 2–3 weeks of absence switched to the 3–4 weeks category, thereby artificially inflating it. Unfortunately, we do not know how many participants originally fell into the 2–3 weeks category and how they handled the gap in the scale.

Our estimates of the outcome are also not corrected for the possibility of varying degrees of employment. In individuals with part-time employment, a temporary inability to work has a higher chance of falling on a work-free time period, leading to underestimation of outcome levels compared with someone with full-time employment. Furthermore, it is known that levels of absence can vary among different professions (17), which was not accounted for in this study.

A further serious limitation is the fact that all measures are subjective reports, potentially affected by biased recall. In particular, there is no objective measure of absence from work, such as physical or electronic documentation. Also, since absences shorter than 3 days do not have to be officially registered with the employer, they may be subject to even stronger forgetting. However, it is unclear whether this would be unevenly distributed among participants in our study.

Personality data were only available for two years (1988, 1993). We imputed the missing years by first averaging the two existing values and imputing them to the remaining years. This approach may gain some validity by following the common assumption that personality consist of largely static traits of an individual. However, even the two values we did record in our study showed some intraindividual variability across the 5-year interval, with correlations for the three subscales being between 0.6 and 0.8.

Finally, fluctuations in economy can also shift rates of absence up and down, with worse economic conditions typically associated with fewer absences (17). This effect was not accounted for in our study.

In conclusion, duration of absence from work was found to be associated with level of psychopathology, older age, being divorced, separated or widowed, and the personality trait of extraversion, but an association with sex, the quality of relationships at and outside of work and other indicators of non-occupational functioning could not be confirmed in this community sample. The discrepancy with the negative finding of Baron and Linden (4) regarding psychopathology could be due to our sample containing fewer cases of chronic disease, but other reasons such as a lack of statistical power are also plausible. Our results, even though tentative, suggest a possible influence of psychopathology on work participation. Therefore, we recommend that insurance medical examinations of work ability include assessments of client’s psychopathological burden in order not to miss potentially important information (18). While the cost of this approach is the waste of resources needed to include a psychopathology assessment if psychopathology in reality has no effect on work ability, we believe these costs to be relatively small, mostly consisting in the inclusion of an additional questionnaire. The benefit, on the other hand, is to not miss a valid predictor of work ability if psychopathology *does* have an effect on work ability, thereby increasing the accuracy of assessment and reducing the cost from inaccurate assessment, adding to our understanding of the determinants of work ability, and providing more targeted care.

## Ethics Statement

All participants provided written informed consent. The study was approved by the institutional review board of the University of Zurich.

## Author Contributions

AG, RS, IW, and ML were responsible for conceiving and designing the study and contributed to data interpretation. AG and IW conducted the data analysis. VA-G, WR, and JA were responsible for data acquisition and contributed to its interpretation. AG wrote the manuscript, and all the co-authors revised its content. All the authors approved the final version of the manuscript and agreed to be held accountable for all aspects of the work.

## Conflict of Interest Statement

WR has been the chief editor of the present journal (*Frontiers in Psychiatry—Public Mental Health*) for the past 3 years. He was not involved in any aspect of the processing of this manuscript. The other authors have no conflicts of interest to declare.
