# Supplementary material for: Duration of Absence from Work Is Related to Psychopathology, Personality, and Sociodemographic Variables in a Longitudinal Cohort
Source: Front Psychiatry. 2017 Nov 29;8:252. doi: 10.3389/fpsyt.2017.00252 (PMC5712568; doi:10.3389/fpsyt.2017.00252)
Supplement: Supplementary file 1 [file data_sheet_1.docx]

***Supplementary Material***

**Duration of absence from work is related to psychopathology, personality, and socio-demographic variables in a longitudinal cohort**

Alex Gamma*, Roman Schleifer, Ingeborg Warnke, Vladeta Ajdacic-Gross, Wulf Rössler, Jules Angst, Michael Liebrenz

*** Correspondence**: Alex Gamma: alexander.gamma@fpd.unibe.ch

## Supplementary Figures and Tables

## Supplementary Figures

- Figure S1: Model 1 (Main model): coefficient plot
- Figure S2: Model 1 (Main model): margins plot
- Figure S3: Model 2 (incl. 1988 data): coefficient plot
- Figure S4: Model 2 (incl. 1988 data): margins plot
- Figure S5: Model 3 (only GSI & Year) : coefficient plot
- Figure S6: Model 3 (only GSI & Year) : margins plot
- Figure S7: Model 4 (no FPI) : coefficient plot
- Figure S8: Model 4 (no FPI) : margins plot
- Figure S9: Model 5 (all outcome categories) : coefficient plot
- ~~Figure S10: Model 5 (all outcome categories) : margins plot:~~ *non-computable*

## Supplementary Tables

- Table S1 Regression coefficients (odds ratios) for models 1- 4
- Table S2 Regression coefficients (odds ratios) for model 5

The margins plot for model 5 is missing because it was not computable due to a nonsymmetric or highly singular variance matrix.

Figure S1: Model 1: Main model: coefficient plot

Figure S2: Model 1: Main model: margins plot

Figure S3: Model 2 (incl. 1988 data) : coefficient plot

Figure S4: Model 2 (incl. 1988 data) : margins plot

Figure S5: Model 3 (only GSI & Year) : coefficient plot

Figure S6: Model 3 (only GSI & Year) : margins plot

Figure S7: Model 4 (no FPI) : coefficient plot

Figure S8: Model 4 (no FPI) : margins plot

**Table S1.** Regression coefficients (odds ratios) for models 1- 4

| **Outcome: Duration of absence from work (3 levels)** | **Main model** | | **Model 2**  **(incl. 1988 data)** | | **Model 3**  **(only GSI & Year)** | | **Model 4**  **(no FPI)** | |
| --- | --- | --- | --- | --- | --- | --- | --- | --- |
| *1-14 days vs no absence* | *OR* | *[95% CI]* | *OR* | *[95% CI]* | *OR* | *[95% CI]* | *OR* | *[95% CI]* |
| Male | 0.99 | [0.67,1.45] | 0.94 | [0.75,1.17] |  |  | 0.89 | [0.63,1.26] |
| GSI | 0.99 | [0.77,1.28] | 1.03 | [0.82,1.29] | 1.14 | [0.93,1.38] | 1.14 | [0.93,1.41] |
| *Year* |  |  |  |  |  |  |  |  |
| 1981 | 0.91 | [0.48,1.75] | 0.88 | [0.59,1.30] | 0.89 | [0.63,1.26] | 1.15 | [0.64,2.08] |
| 1986 | 0.33* | [0.13,0.83] | 0.35*** | [0.23,0.54] | 0.37*** | [0.25,0.55] | 0.42* | [0.18,0.96] |
| 1988 |  |  | 2.52*** | [1.70,3.73] | 2.52*** | [1.79,3.53] |  |  |
| 1993 | 1.15 | [0.55,2.41] | 1.51+ | [1.00,2.29] | 1.45* | [1.03,2.04] | 1.3 | [0.64,2.63] |
| 1999 | 3.08* | [1.25,7.59] | 1.83** | [1.19,2.82] | 1.83*** | [1.30,2.58] | 3.97** | [1.64,9.58] |
| *Year X GSI* |  |  |  |  |  |  |  |  |
| 1981 X GSI | 1.02 | [0.61,1.72] | 1.23 | [0.87,1.74] | 1.18 | [0.87,1.58] | 0.82 | [0.52,1.29] |
| 1986 X GSI | 1.59 | [0.78,3.24] | 1.27 | [0.87,1.85] | 1.28 | [0.91,1.80] | 1.45 | [0.81,2.61] |
| 1988 X GSI |  |  | 0.9 | [0.63,1.27] | 0.91 | [0.65,1.26] |  |  |
| 1993 X GSI | 1.11 | [0.65,1.89] | 1.15 | [0.82,1.63] | 1.17 | [0.84,1.62] | 1.15 | [0.68,1.92] |
| 1999 X GSI | 0.78 | [0.36,1.72] | 0.98 | [0.68,1.43] | 0.96 | [0.67,1.37] | 0.78 | [0.36,1.68] |
| *FPI (std)* |  |  |  |  |  |  |  |  |
| Aggressiveness | 1.08 | [0.87,1.34] | 1.19** | [1.05,1.35] |  |  |  |  |
| Extraversion | 1.19 | [0.96,1.48] | 1.14* | [1.00,1.28] |  |  |  |  |
| Neuroticism/vegetative lability | 1.35* | [1.06,1.71] | 1.09 | [0.93,1.26] |  |  |  |  |
| Conflicts at work | 0.95 | [0.59,1.52] | 1.23+ | [0.97,1.57] |  |  | 0.87 | [0.57,1.33] |
| *Marital status* |  |  |  |  |  |  |  |  |
| Married | 2.73* | [1.03,7.25] | 0.75* | [0.57,1.00] |  |  | 2.54* | [1.09,5.95] |
| Divorced/separated/widowed | 1.41 | [0.58,3.43] | 1.42 | [0.88,2.31] |  |  | 1.22 | [0.51,2.92] |
| *Conflicts with friends* |  |  |  |  |  |  |  |  |
| have occurred | 1.62* | [1.07,2.47] |  |  |  |  | 1.59* | [1.09,2.32] |
| occasional | 2.40* | [1.02,5.66] |  |  |  |  | 2.24* | [1.09,4.58] |
| frequent | 2 | [0.42,9.41] |  |  |  |  | 2.28 | [0.55,9.49] |
| termination of relationship | 0.94 | [0.19,4.72] |  |  |  |  | 0.86 | [0.19,3.98] |
| *Conflicts with partner* |  |  |  |  |  |  |  |  |
| have occurred | 1.31 | [0.74,2.33] |  |  |  |  | 0.99 | [0.59,1.65] |
| occasional | 1.02 | [0.47,2.20] |  |  |  |  | 0.93 | [0.46,1.87] |
| frequent | 0.67 | [0.14,3.19] |  |  |  |  | 0.55 | [0.12,2.46] |
| termination of relationship | 3.59* | [1.04,12.42] |  |  |  |  | 3.20* | [1.07,9.58] |
| Increasing difficulties with partner | 0.8 | [0.51,1.26] | 0.98 | [0.75,1.28] |  |  | 0.76 | [0.50,1.14] |
| Improvement of relationship with partner | 0.7 | [0.41,1.19] | 0.91 | [0.70,1.18] |  |  | 0.71 | [0.45,1.13] |
| Number of meetings with opposite sex | 0.93 | [0.79,1.09] |  |  |  |  | 0.87+ | [0.75,1.00] |
| Lives alone | 1.02 | [0.64,1.61] | 0.99 | [0.75,1.31] |  |  | 0.93 | [0.60,1.42] |
| Number of close friends | 0.91 | [0.75,1.12] |  |  |  |  | 0.88 | [0.73,1.06] |
| Jobless | 1.14 | [0.45,2.90] | 0.76 | [0.44,1.29] |  |  | 0.95 | [0.40,2.30] |
|  |  |  |  |  |  |  |  |  |
| *≥ 3 weeks vs no absence* | *OR* | *[95% CI]* | *OR* | *[95% CI]* | *OR* | *[95% CI]* | *OR* | *[95% CI]* |
| Male | 1.31 | [0.55,3.11] | 1.09 | [0.72,1.66] |  |  | 1.6 | [0.72,3.53] |
| GSI | 1.71+ | [0.97,3.01] | 1.35 | [0.81,2.25] | 1.45+ | [0.94,2.23] | 1.71* | [1.07,2.73] |
| *Year* |  |  |  |  |  |  |  |  |
| 1981 | 1.67 | [0.44,6.36] | 0.98 | [0.35,2.76] | 1.27 | [0.52,3.12] | 1.49 | [0.43,5.18] |
| 1986 | 0.08 | [0.00,2.94] | 0.63 | [0.21,1.86] | 0.77 | [0.29,2.02] | 0.64 | [0.10,4.01] |
| 1988 |  |  | 5.09*** | [2.06,12.57] | 5.49*** | [2.50,12.09] |  |  |
| 1993 | 1.75 | [0.28,10.83] | 4.14** | [1.63,10.50] | 4.43*** | [2.02,9.68] | 1.41 | [0.25,8.09] |
| 1999 | 1.96 | [0.15,25.35] | 1.7 | [0.59,4.92] | 2.18+ | [0.89,5.34] | 1.46 | [0.13,16.73] |
| *Year X GSI* |  |  |  |  |  |  |  |  |
| 1981 X GSI | 1.48 | [0.68,3.25] | 1.84+ | [0.95,3.58] | 1.36 | [0.77,2.41] | 1.18 | [0.58,2.40] |
| 1986 X GSI | 5.22+ | [0.79,34.29] | 1.23 | [0.57,2.65] | 1.06 | [0.52,2.16] | 1.09 | [0.34,3.51] |
| 1988 X GSI |  |  | 1.11 | [0.59,2.09] | 1.09 | [0.62,1.92] |  |  |
| 1993 X GSI | 1.91 | [0.69,5.33] | 0.87 | [0.45,1.68] | 0.88 | [0.48,1.61] | 1.64 | [0.62,4.31] |
| 1999 X GSI | 1.87 | [0.37,9.52] | 1.2 | [0.55,2.58] | 1.07 | [0.53,2.17] | 1.75 | [0.35,8.81] |
| *FPI (std)* |  |  |  |  |  |  |  |  |
| Aggressiveness | 1 | [0.63,1.58] | 1.06 | [0.85,1.33] |  |  |  |  |
| Extraversion | 1.66* | [1.03,2.67] | 1.61*** | [1.28,2.03] |  |  |  |  |
| Neuroticism/vegetative lability | 1.04 | [0.61,1.77] | 1.28+ | [0.97,1.69] |  |  |  |  |
| Conflicts at work | 0.63 | [0.21,1.86] | 1.53+ | [0.98,2.37] |  |  | 0.77 | [0.31,1.92] |
| *Marital status* |  |  |  |  |  |  |  |  |
| Married | 0 | [0.00,.] | 0.75 | [0.45,1.24] |  |  | 1.94 | [0.20,18.68] |
| Divorced/separated/widowed | 3.49 | [0.64,18.94] | 2.93** | [1.44,5.95] |  |  | 3.65 | [0.77,17.36] |
| *Conflicts with friends* |  |  |  |  |  |  |  |  |
| have occurred | 0.79 | [0.30,2.09] |  |  |  |  | 0.87 | [0.36,2.08] |
| occasional | 1.82 | [0.41,8.08] |  |  |  |  | 2.07 | [0.59,7.29] |
| frequent | 0 | [0.00,.] |  |  |  |  | 0 | [0.00,.] |
| termination of relationship | 0 | [0.00,.] |  |  |  |  | 0 | [0.00,.] |
| *Conflicts with partner* |  |  |  |  |  |  |  |  |
| have occurred | 1.31 | [0.35,4.91] |  |  |  |  | 1.31 | [0.41,4.21] |
| occasional | 1.54 | [0.28,8.33] |  |  |  |  | 1.08 | [0.21,5.56] |
| frequent | 0 | [0.00,.] |  |  |  |  | 0 | [0.00,.] |
| termination of relationship | 3.64 | [0.48,27.43] |  |  |  |  | 2.65 | [0.38,18.27] |
| Increasing difficulties with partner | 1.08 | [0.43,2.70] | 0.68 | [0.40,1.18] |  |  | 1.06 | [0.46,2.45] |
| Improvement of relationship with partner | 1.48 | [0.45,4.83] | 1.09 | [0.66,1.81] |  |  | 0.99 | [0.32,3.02] |
| Number of meetings with opposite sex | 1.18 | [0.84,1.65] |  |  |  |  | 1.07 | [0.80,1.43] |
| Lives alone | 0.85 | [0.32,2.26] | 0.79 | [0.46,1.36] |  |  | 0.79 | [0.32,1.95] |
| Number of close friends | 0.69+ | [0.44,1.07] |  |  |  |  | 0.67* | [0.45,0.98] |
| Jobless | 1.4 | [0.25,7.88] | 1.73 | [0.82,3.62] |  |  | 1.35 | [0.26,6.90] |

+ p<0.10, * p<0.05, ** p<0.01, *** p<0.001

Figure S9: Model 5 (all outcome categories): coefficient plot

**Table S2.** Regression coefficients (odds ratios) for model 5

| **Outcome: Duration of absence from work (6 levels)** | **1-2** **days vs no absence** | | **3-6 days vs no absence** | | **1-2** **weeks vs no absence** | | **3-4 weeks vs no absence** | | **>4 weeks vs no absence** | |
| --- | --- | --- | --- | --- | --- | --- | --- | --- | --- | --- |
|  | *OR* | *[95% CI]* | *OR* | *[95% CI]* | *OR* | *[95% CI]* | *OR* | *[95% CI]* | *OR* | *[95% CI]* |
| Male | 1.13 | [0.69,1.88] | 0.84 | [0.50,1.43] | 0.94 | [0.43,2.05] | 1.68 | [0.58,4.87] | 1.11 | [0.23,5.41] |
| GSI | 1.02 | [0.74,1.41] | 1.01 | [0.71,1.44] | 0.87 | [0.48,1.60] | 2.18* | [1.14,4.17] | 0.77 | [0.17,3.40] |
| Year |  |  |  |  |  |  |  |  |  |  |
| 1981 | 0.68 | [0.28,1.66] | 1.3 | [0.53,3.19] | 0.75 | [0.18,3.20] | 3.42 | [0.68,17.11] | 0.23 | [0.01,3.59] |
| 1986 | 0.38+ | [0.12,1.17] | 0.19+ | [0.03,1.25] | 0.31 | [0.04,2.38] | 0.61 | [0.03,11.31] | 0 | [0.00,.] |
| 1988 |  |  |  |  |  |  |  |  |  |  |
| 1993 | 0.55 | [0.19,1.58] | 1.83 | [0.68,4.94] | 2.53 | [0.63,10.27] | 2.42 | [0.24,24.68] | 0.55 | [0.02,12.07] |
| 1999 | 1.3 | [0.39,4.38] | 7.42*** | [2.48,22.23] | 2.76 | [0.51,14.95] | 2 | [0.08,50.76] | 0.69 | [0.01,37.00] |
| Year X GSI |  |  |  |  |  |  |  |  |  |  |
| 1981 X GSI | 0.81 | [0.36,1.84] | 1.07 | [0.55,2.10] | 1.35 | [0.50,3.66] | 1.17 | [0.49,2.78] | 3.29 | [0.53,20.56] |
| 1986 X GSI | 1.58 | [0.63,3.95] | 2.26 | [0.63,8.10] | 1.53 | [0.31,7.50] | 0.7 | [0.04,11.87] | 3.43E+37 | [0.00,.] |
| 1988 X GSI |  |  |  |  |  |  |  |  |  |  |
| 1993 X GSI | 1.11 | [0.55,2.27] | 0.93 | [0.44,1.99] | 1.5 | [0.62,3.62] | 1.39 | [0.40,4.85] | 4.71 | [0.69,32.34] |
| 1999 X GSI | 0.63 | [0.19,2.08] | 0.75 | [0.29,1.89] | 1.14 | [0.29,4.49] | 0.8 | [0.04,14.70] | 4.28 | [0.37,49.64] |
| FPI (std) |  |  |  |  |  |  |  |  |  |  |
| Aggressiveness | 1.33* | [1.01,1.74] | 0.93 | [0.69,1.26] | 0.79 | [0.51,1.23] | 0.98 | [0.57,1.69] | 1.05 | [0.43,2.55] |
| Extraversion | 1.31+ | [0.98,1.73] | 0.98 | [0.74,1.31] | 1.46+ | [0.94,2.26] | 1.83* | [1.04,3.20] | 1.28 | [0.54,3.03] |
| Neuroticism/vegetative lability | 1.26 | [0.92,1.71] | 1.31 | [0.95,1.81] | 1.71* | [1.05,2.78] | 1.13 | [0.59,2.15] | 1 | [0.38,2.63] |
| Conflicts at work | 1.08 | [0.59,1.99] | 0.69 | [0.35,1.37] | 1.44 | [0.60,3.44] | 0.33 | [0.07,1.61] | 1.33 | [0.21,8.28] |
| Marital status |  |  |  |  |  |  |  |  |  |  |
| Married | 2.99+ | [0.83,10.84] | 3.01+ | [0.94,9.67] | 1.63 | [0.27,9.86] | 0 | [0.00,.] | 0 | [0.00,.] |
| Divorced/separated/widowed | 1.53 | [0.46,5.06] | 0.99 | [0.31,3.14] | 2.1 | [0.50,8.84] | 6.30+ | [0.77,51.72] | 2.64 | [0.16,44.23] |
| Conflicts with friends |  |  |  |  |  |  |  |  |  |  |
| have occurred | 1.47 | [0.85,2.55] | 2.12** | [1.21,3.71] | 1.03 | [0.43,2.45] | 0.83 | [0.26,2.58] | 0.35 | [0.04,3.40] |
| occasional | 2.57+ | [0.84,7.79] | 2.91+ | [0.97,8.71] | 1.46 | [0.31,6.82] | 1.29 | [0.19,8.94] | 2.61 | [0.26,25.82] |
| frequent | 1.32 | [0.13,13.52] | 3.64 | [0.64,20.57] | 0 | [0.00,.] | 0 | [0.00,.] | 0 | [0.00,.] |
| termination of relationship | 1.39 | [0.20,9.88] | 0.76 | [0.07,8.56] | 0.83 | [0.06,10.75] | 0 | [0.00,.] | 0 | [0.00,.] |
| Conflicts with partner |  |  |  |  |  |  |  |  |  |  |
| have occurred | 0.89 | [0.41,1.91] | 1.33 | [0.61,2.89] | 4.28* | [1.31,13.98] | 2.09 | [0.49,8.94] | 0 | [0.00,.] |
| occasional | 0.55 | [0.18,1.68] | 1.21 | [0.45,3.26] | 3.4 | [0.75,15.48] | 2.74 | [0.47,16.01] | 0 | [0.00,.] |
| frequent | 0.37 | [0.04,3.51] | 0.51 | [0.05,5.35] | 6.07 | [0.50,74.36] | 0 | [0.00,.] | 0 | [0.00,.] |
| termination of relationship | 1.38 | [0.21,9.06] | 2.69 | [0.51,14.10] | 23.28*** | [4.20,129.13] | 2.88 | [0.22,37.26] | 8.83 | [0.42,184.04] |
| Increasing difficulties with partner | 0.52+ | [0.27,1.00] | 1.19 | [0.66,2.14] | 0.86 | [0.33,2.23] | 0.91 | [0.29,2.88] | 1.4 | [0.27,7.26] |
| Improvement of relationship with partner | 0.58 | [0.28,1.19] | 0.77 | [0.38,1.59] | 0.89 | [0.28,2.78] | 1.97 | [0.51,7.61] | 0.91 | [0.07,12.64] |
| Number of meetings with opposite sex | 0.87 | [0.71,1.07] | 0.94 | [0.75,1.17] | 1.17 | [0.84,1.63] | 1.23 | [0.81,1.85] | 1.17 | [0.66,2.08] |
| Lives alone | 1.05 | [0.56,1.96] | 0.8 | [0.42,1.52] | 1.98 | [0.83,4.73] | 0.87 | [0.27,2.83] | 1.29 | [0.20,8.26] |
| Number of close friends | 0.94 | [0.72,1.22] | 0.85 | [0.64,1.12] | 0.97 | [0.64,1.46] | 0.84 | [0.49,1.44] | 0.46+ | [0.21,1.01] |
| Jobless | 1.77 | [0.54,5.77] | 0.88 | [0.24,3.26] | 0.73 | [0.12,4.43] | 1.05 | [0.10,10.80] | 3.81 | [0.31,46.50] |
